# Human Papillomavirus Vaccination Estimates Among Adolescents in the Mississippi Delta Region: National Immunization Survey‑Teen, 2015–2017

**DOI:** 10.5888/pcd17.190234

**Published:** 2020-04-16

**Authors:** David Yankey, Laurie D. Elam-Evans, Connie L. Bish, Shannon K. Stokley

**Affiliations:** 1 National Center for Immunization and Respiratory Diseases, Centers for Disease Control and Prevention, Atlanta, Georgia; 2 National Center for Chronic Disease Prevention and Health Promotion, Centers for Disease Control and Prevention, Atlanta, Georgia

## Abstract

**Introduction:**

The Delta Regional Authority (DRA) consists of 252 counties and parishes in 8 states in the US Mississippi Delta region. DRA areas have high rates of disease, including cancers related to the human papillomavirus (HPV). HPV vaccination coverage in the DRA region has not been documented.

**Methods:**

We analyzed data for 63,299 adolescents aged 13 to 17 years in the National Immunization Survey-Teen, 2015–2017. We compared HPV vaccination initiation coverage estimates (≥1 dose) in the DRA region with coverage estimates in areas in the 8 Delta states outside the DRA region and non-Delta states. We examined correlates of HPV vaccination coverage initiation and reasons parents did not intend to vaccinate adolescents.

**Results:**

Vaccination rates in the DRA region (n = 2,317; 54.3%) and in Delta areas outside the DRA region (n = 6,028; 56.2%) were similar, but these rates were significantly lower than rates in non-Delta states (n = 54,954; 61.4%). Inside the DRA region, reasons for parents’ vaccine hesitancy or refusal were similar to those expressed by parents in the Delta areas outside the DRA region. Some parents believed that the vaccine was not necessary or had concerns about vaccine safety.

**Conclusion:**

HPV vaccination coverage in the DRA region is similar to coverage in other Delta counties and parishes, but it is significantly lower than in non-Delta states. Activities to address parental concerns and improve provider recommendations for the vaccine in the DRA region are needed to increase HPV vaccination rates.

**Summary**

**What is already known about this topic?**

Routine human papillomavirus (HPV) vaccination is recommended for children aged 11 or 12 years to prevent HPV and associated cancers.

**What is added by this report?**

Geographic disparities in HPV vaccine coverage exist in the Mississippi Delta Regional Authority (DRA) counties and other counties in Delta states, compared with states outside the Delta region.

**What are the implications for public health practice?**

Efforts to improve coverage are needed, particularly in the DRA region and other counties in Delta states. Providing parents and guardians with information and strong, compelling recommendations can improve HPV vaccination coverage.

## Introduction

The Delta Regional Authority (DRA) was established in 2000 by the US Congress to support economic development and improve living standards for approximately 10 million residents in 252 designated counties and parishes in 8 Mississippi Delta states: Alabama, Arkansas, Illinois, Kentucky, Louisiana, Mississippi, Missouri, and Tennessee (Figure) (1). Counties in the DRA region are significantly disadvantaged, and 43.3% are classified as in persistent poverty, versus 11.2% in the nation as a whole (2).

**Figure:**
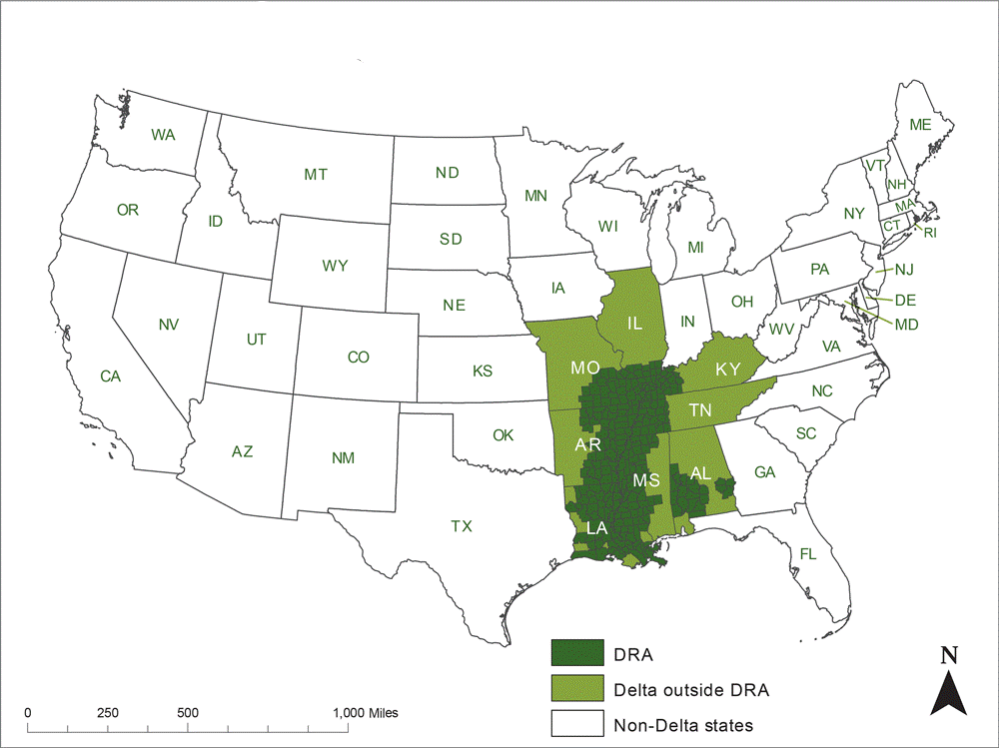
United States’ Delta Regional Authority (DRA) counties and parishes.

Residents of the Delta region have a poorer health status than do other US residents (3). Residents in the DRA region are more likely than other US residents to have a high body mass index, high blood pressure, diabetes, and are more likely to smoke and die of cancer (4,5). Although DRA counties are uniquely disadvantaged, they are located in Mississippi Delta states where other counties have similar demographic factors. Despite similarities, compared with counties in these states but outside the DRA region, DRA counties have substantially worse health indicators, such as those associated with social determinants of health and cardiovascular disease morbidity and mortality (6,7).

A 2005 county-level analysis indicated that DRA counties had a median cancer mortality rate that was approximately 8.5% higher than in counties in Delta states outside the DRA region (8). Overall, residents inside DRA areas have a substantially higher incidence of human papilloma virus (HPV)-associated cancers than US residents overall (9), particularly cervical cancer (10), as well as a higher incidence of sexually transmitted infections other than HPV (11).

The Advisory Committee on Immunization Practices (ACIP) recommends routine vaccination of children aged 11 or 12 years with HPV vaccine to prevent HPV-associated cancers (12,13). Despite this recommendation, HPV vaccination coverage has remained low nationally relative to other recommended vaccines (14). Coverage of HPV vaccine in DRA areas has not been documented.

The objective of this analysis was to 1) better understand HPV vaccination coverage among adolescents in DRA areas and how it compares to the rest of the nation, 2) examine the association of sociodemographic and health care-related factors with HPV vaccination, and 3) examine HPV vaccination intentions and reasons for hesitancy among parents of unvaccinated adolescents.

## Methods

The National Immunization Survey-Teen (NIS-Teen) is a random-digit–dialed telephone survey of parents or guardians of adolescents aged 13 to 17 years. NIS-Teen also includes a survey mailed to all vaccination providers identified by the parent and those who consented to contact for vaccination history (15). NIS-Teen uses a national probability sample of households in the United States, which includes all 50 states, the District of Columbia, and selected local areas.

We analyzed NIS-Teen data from 2015, 2016, and 2017, collected from households by way of landlines and cellular telephones (16,17). Provider-reported vaccination records were used to determine all HPV vaccination coverage estimates among adolescents. In this analysis, adolescents without adequate provider data were excluded; NIS-Teen methodology assigns provider phase weights to control for both provider nonresponse and for adolescents without adequate provider data for other reasons (18,19). Details of the NIS-Teen methodology, including how multiple survey years of vaccination data are combined to produce a synthesized immunization history and a description of the weighting procedure, have been published (16,17). The 2015–2017 NIS-Teen was approved by the National Center for Health Statistics Research Ethics Review Board, and the NORC (National Opinion Research Center) at the University of Chicago Institutional Review Board.

We included data from 63,299 adolescents, aged 13 to 17 years, in 2015–2017 NIS-Teen. Inclusion criteria required that adolescents have adequate provider data (ie, vaccination history documentation from provider reports) to determine whether they were up-to-date with vaccinations. The Council of American Survey Research Organizations (CASRO) landline response rates were 56.4% for 2015, 55.5% for 2016, and 51.5% for 2017. CASRO response rates for the cell phone sample were 29.8% for 2015, 29.5% for 2016, and 23.5% for 2017 (20). The annual number of adolescents with completed household interviews and adequate provider data in the sample was 21,875 (49.8%) for 2015; 20,475 (48.8%) for 2016; and 20,949 (48.1%) for 2017 (20).

We categorized our study population into 3 geographic areas: inside DRA areas (n = 2,317), Delta areas outside the DRA (counties and parishes outside the DRA in the 8 Mississippi Delta states, n = 6,028), and non-Delta states (the District of Columbia and the remaining United States that are outside the Mississippi Delta, n = 54,954). We examined a dichotomous outcome of HPV vaccine initiation (≥1 HPV vaccine dose or not vaccinated). We compared HPV vaccination initiation coverage estimates in the DRA region to coverage estimates in the other 2 geographic areas. We also estimated HPV vaccination initiation coverage for selected covariates, including demographic characteristics (sex, age, and race/ethnicity of adolescent mother’s education, marital status, income-to-poverty ratio [IPR, total family income divided by the federal poverty level], and residence), health insurance, and access to care variables for adolescents (preventive care visit at 11–12 years, received provider recommendation for HPV, total number of vaccination providers, number of physician contacts in the past year, and type of facility providing the vaccinations). Among parents with adolescents unvaccinated for HPV, we examined intent to vaccinate their adolescents in the next year by asking parents, “How likely is it that [TEEN] will receive HPV shots in the next 12 months?” Response options included “very likely,” “somewhat likely,” “not sure or don’t know,” “not too likely,” and “not likely at all.” Parents who indicated the last 3 responses were asked, “What is the main reason [TEEN] will not receive HPV shots in the next 12 months?” This open-ended question allowed parents to indicate multiple reasons, and we identified the top 5 reasons from verbatim responses.

We conducted statistical analyses by using SAS-callable SUDAAN release 11.0.1 (RTI International) to account for the complex sampling design of the NIS-Teen data. Point estimates and their 95% confidence intervals (CIs) were weighted to be representative of the areas from which the households were sampled. We used bivariate analyses to describe the distribution across selected sociodemographic characteristics. We used *t* tests to identify significant differences (*P* < .05) in the proportion of categories between the DRA region and Delta areas outside the DRA authority and non-Delta states. We also conducted a multivariable logistic regression analysis to produce adjusted prevalence ratios (APRs) and 95% CIs by using a standard statement in SUDAAN procedures to produce such estimates (model-adjusted risk). We used χ^2^ tests to identify covariates that were associated with HPV vaccination initiation in each of the 3 geographic areas.

## Results

Demographic characteristics differed substantially between adolescents living inside DRA areas and adolescents living in Delta areas outside the DRA or in non-Delta states (Table 1). We found a significantly higher proportion of non-Hispanic black adolescents, adolescents living in rural areas, and adolescents living in a household with an IPR below 133% in the DRA region. A significantly smaller proportion of adolescents inside the DRA region had a mother who had graduated from college or was married, and a higher percentage had mothers aged 34 or younger. Adolescents living in the DRA region also differed significantly in health care access and use, and they were more likely to be enrolled in Medicaid, to have had 4 or more physician contacts in the previous year, to have 2 or 3 vaccination providers, to have received all of their vaccinations in public facilities or a mix of facilities, and to be less likely to report receiving a provider recommendation for the HPV vaccination.

**Table 1:** Characteristics of Adolescents Aged 13–17 Years by Geographic Area, National Immunization Survey–Teen, United States, 2015–2017

Characteristic	All Surveyed Adolescents in the United States		Mississippi Delta States				Non-Delta States	
			DRA Counties		Delta Areas Outside DRA			
	Sample Size	Weighted% (95% CI)	Sample Size	Weighted% (95% CI)	Sample Size	Weighted% (95% CI)	Sample Size	Weighted% (95% CI)
**Overall**	63,299	100.0 (—)	2,317	3.0 (2.8–3.1)	6,028	9.9 (9.7–10.1)	54,954	87.1 (86.9–87.3)
**Interview year**	63,299	100.0 (—)	2,317	100.0 (—)	6,028	100.0 (—)	54,954	100.0 (—)
2015	21,875	33.3 (32.9–33.8)	819	34.9 (32.9–37.0)	2,060	33.2 (32.3–34.1)	18,996	33.3 (32.8–33.8)
2016	20,475	33.3 (32.9–33.8)	771	33.8 (31.8–35.9)	1,907	33.1 (32.2–34.1)	17,797	33.3 (32.9–33.8)
2017	20,949	33.3 (32.9–33.8)^a^	727	31.3 (29.2–33.3)	2,061	33.7 (32.7–34.6)	18,161	33.4 (32.9–33.9)^a^
**Sex**	63,299	100.0 (—)	2,317	100.0 (—)	6,028	100.0 (—)	54,954	100.0 (—)
Male	33,285	51.1 (50.3–51.8)	1,258	51.9 (49.3–54.5)	3,142	50.8 (49.2–52.4)	28,885	51.1 (50.2–51.9)
Female	30,014	48.9 (48.2–49.7)	1,059	48.1 (45.5–50.7)	2,886	49.2 (47.6–50.8)	26,069	48.9 (48.1–49.8)
**Age of adolescent at interview, y**	63,299	100.0 (—)	2,317	100.0 (—)	6,028	100.0 (—)	54,954	100.0 (—)
13	12,968	19.8 (19.2–20.3)	498	21.6 (19.6–23.8)	1,228	19.9 (18.6–21.2)	11,242	19.7 (19.0–20.3)
14	13,252	19.9 (19.3–20.5)	453	19.5 (17.5–21.7)	1,206	19.1 (17.9–20.4)	11,593	20.0 (19.3–20.7)
15	12,770	21.0 (20.4–21.6)	474	20.9 (18.9–23.1)	1,251	20.6 (19.4–21.9)	11,045	21.0 (20.4–21.7)
16	12,811	20.5 (19.9–21.1)^a^	457	18.2 (16.4–20.2)	1,270	21.4 (20.1–22.7)^a^	11,084	20.5 (19.8–21.1)^a^
17	11,498	18.9 (18.3–19.4)	435	19.7 (17.7–21.9)	1,073	19.0 (17.7–20.3)	9,990	18.8 (18.2–19.5)
**Adolescent’s race/ethnicity**	63,299	100.0 (—)	2,317	100.0 (—)	6,028	100.0 (—)	54,954	100.0 (—)
Non-Hispanic white	38,728	52.9 (52.1–53.6)	1,322	51.0 (48.4–53.5)	3,959	63.5 (61.9–65.0)^a^	33,447	51.7 (50.9–52.5)
Non-Hispanic black	5,961	13.9 (13.4–14.4)^b^	700	38.2 (35.6–40.8)	743	14.9 (13.7–16.1)^b^	4,518	12.9 (12.4–13.5)^b^
Hispanic	11,715	23.2 (22.5–23.9)^a^	148	4.8 (4.0–5.9)	834	13.3 (12.3–14.4)^a^	10,733	25.0 (24.2–25.8)^a^
Other	6,895	10.1 (9.6–10.5)^a^	147	6.0 (5.0–7.3)	492	8.4 (7.5–9.3)^a^	6,256	10.4 (9.9–10.9)^a^
**Mother’s education**	63,299	100.0 (—)	2,317	100.0 (—)	6,028	100.0 (—)	54,954	100.0 (—)
<High school graduate	7,725	13.3 (12.8–13.9)	335	12.0 (10.5–13.6)	830	10.8 (9.9–11.7)	6,560	13.7 (13.0–14.3)^a^
High school graduate	10,020	22.5 (21.8–23.2)^b^	497	28.9 (26.5–31.5)	1,070	23.6 (22.1–25.1)^b^	8,453	22.1 (21.4–22.9)^b^
>High school graduate, some college	16,311	24.9 (24.2–25.5)^b^	694	30.8 (28.5–33.2)	1,647	26.9 (25.5–28.3)^b^	13,970	24.4 (23.7–25.1)^b^
College graduate	29,243	39.3 (38.6–40.0)^a^	791	28.3 (26.2–30.5)	2,481	38.8 (37.2–40.3)^a^	25,971	39.8 (39.0–40.5)^a^
**Mother’s marital status**	59,126	100.0 (—)	2,136	100.0 (—)	5,617	100.0 (—)	51,373	100.0 (—)
Married	44,381	67.7 (66.9–68.4)^a^	1,348	51.9 (49.1–54.6)	4,050	65.5 (63.8–67.1)^a^	38,983	68.4 (67.6–69.3)^a^
Divorced, widowed, or separated	10,729	23.3 (22.6–24.0)^b^	511	30.2 (27.6–32.8)	1,120	24.4 (23.0–26.0)^b^	9,098	22.9 (22.2–23.7)^b^
Never married	4,016	9.1 (8.6–9.5)^b^	277	18.0 (15.8–20.4)	447	10.1 (9.1–11.2)^b^	3,292	8.6 (8.1–9.1)^b^
**Mother’s age, y**	63,299	100.0 (—)	2,317	100.0 (—)	6,028	100.0 (—)	54,954	100.0 (—)
≤34	5,151	8.8 (8.4–9.2)^b^	303	14.5 (12.7–16.4)	575	9.6 (8.7–10.6)^b^	4,273	8.5 (8.0–9.0)^b^
35–44	25,998	43.7 (42.9–44.4)^b^	1,072	49.0 (46.4–51.5)	2,688	46.2 (44.6–47.8)	22,238	43.2 (42.4–44.0)^b^
≥45	32,150	47.5 (46.8–48.3)^a^	942	36.6 (34.2–39.1)	2,765	44.2 (42.6–45.8)^a^	28,443	48.3 (47.5–49.1)^a^
**Adolescent had preventive care visit at age 11 or 12**	62,875	100.0 (—)	2,299	100.0 (—)	6,000	100.0 (—)	54,576	100.0 (—)
Yes	57,452	90.9 (90.4–91.3)	2,053	89.7 (88.1–91.2)	5,476	90.7 (89.7–91.6)	49,923	91.0 (90.4–91.4)
No	5,423	9.1 (8.7–9.6)	246	10.3 (8.8–11.9)	524	9.3 (8.4–10.3)	4,653	9.0 (8.6–9.6)
**Income-to-poverty ratio**	63,299	100.0 (—)	2,317	100.0 (—)	6,028	100.0 (—)	54,954	100.0 (—)
<133%	16,687	32.0 (31.3–32.8)^b^	869	42.6 (40.0–45.2)	1,837	32.8 (31.3–34.4)^b^	13,981	31.6 (30.8–32.4)^b^
133% to <322%	17,243	28.1 (27.4–28.7)	665	30.4 (28.0–32.9)	1,754	30.9 (29.4–32.4)	14,824	27.7 (26.9–28.4)^b^
322% to <503%	13,132	18.2 (17.7–18.7)^a^	387	14.0 (12.5–15.8)	1,183	18.2 (17.0–19.4)^a^	11,562	18.3 (17.7–18.9)^a^
≥503%	16,237	21.7 (21.1–22.3)^a^	396	13.0 (11.6–14.6)	1,254	18.1 (17.0–19.3)^a^	14,587	22.4 (21.8–23.1)^a^
**Health insurance status^c^**	63,299	100.0 (—)	2,317	100.0 (—)	6,028	100.0 (—)	54,954	100.0 (—)
Private only	36,269	51.6 (50.8–52.3)^a^	1,014	37.9 (35.5–40.3)	3,302	52.8 (51.2–54.4)^a^	31,953	51.9 (51.1–52.7)^a^
Any Medicaid	19,717	37.1 (36.3–37.8)^b^	1,053	51.8 (49.3–54.4)	2,169	38.5 (36.9–40.0)^b^	16,495	36.4 (35.6–37.2)^b^
Other^d^	4,885	7.1 (6.7–7.4)	164	6.8 (5.6–8.2)	359	4.9 (4.3–5.5)^b^	4,362	7.3 (7.0–7.7)
Uninsured	2,428	4.3 (4.0–4.6)	86	3.5 (2.7–4.6)	198	3.9 (3.2–4.6)	2,144	4.4 (4.0–4.7)
**Provider recommended HPV vaccination**	57,740	100.0 (—)	2,071	100.0 (—)	5,519	100.0 (—)	50,150	100.0 (—)
Yes	42,603	71.7 (71.0–72.4)^a^	1,311	63.5 (60.8–66.0)	3,765	68.4 (66.8–69.9)^a^	37,527	72.4 (71.6–73.2)^a^
No	15,137	28.3 (27.6–29.0)^b^	760	36.5 (34.0–39.2)	1,754	31.6 (30.1–33.2)^b^	12,623	27.6 (26.8–28.4)^b^
**No. of providers**	63,109	100.0 (—)	2,312	100.0 (—)	6,013	100.0 (—)	54,784	100.0 (—)
1	36,114	59.3 (58.6–60.0)^a^	1,201	53.2 (50.6–55.7)	3,379	57.3 (55.7–58.8)^a^	31,534	59.7 (58.9–60.5)^a^
2 or 3	17,345	26.4 (25.7–27.0)^b^	739	32.6 (30.3–35.1)	1,757	29.5 (28.1–31.0)^b^	14,849	25.8 (25.1–26.5)^b^
≥4	9,650	14.3 (13.8–14.9)	372	14.2 (12.5–16.0)	877	13.2 (12.2–14.3)	8,401	14.5 (13.9–15.1)
**No. of physician contacts in the past year**	62,668	100.0 (—)	2,283	100.0 (—)	5,975	100.0 (—)	54,410	100.0 (—)
None	8,356	15.2 (14.6–15.8)^a^	267	12.8 (11.1–14.8)	738	13.4 (12.3–14.6)	7,351	15.5 (14.8–16.1)^a^
1	17,801	30.0 (29.3–30.7)^a^	568	25.6 (23.3–27.9)	1,622	29.1 (27.6–30.6)^a^	15,611	30.3 (29.5–31.1)^a^
2 or 3	22,914	35.2 (34.5–35.9)	852	36.3 (33.9–38.8)	2,275	37.5 (36.0–39.1)	19,787	34.9 (34.1–35.7)
≥4	13,597	19.6 (19.0–20.1)^b^	596	25.3 (23.1–27.6)	1,340	20.0 (18.8–21.3)^b^	11,661	19.3 (18.7–20.0)^b^
**Type of facility where vaccinations were received**	62,872	100.0 (—)	2,301	100.0 (—)	5,990	100.0 (—)	54,581	100.0 (—)
All private	31,374	53.4 (52.7–54.2)^a^	810	36.0 (33.5–38.5)	2,804	47.6 (46.0–49.2)^a^	27,760	54.7 (53.9–55.5)^a^
All public	9,205	14.8 (14.3–15.4)^b^	570	26.1 (23.9–28.5)	1,060	17.3 (16.1–18.5)^b^	7,575	14.1 (13.6–14.8)^b^
All hospital	7,222	9.7 (9.3–10.1)^a^	187	8.2 (6.9–9.8)	565	9.5 (8.6–10.5)	6,470	9.8 (9.3–10.2)^a^
Mixed^e^	13,135	18.9 (18.3–19.4)^b^	677	27.1 (24.9–29.4)	1,401	22.9 (21.6–24.2)^b^	11,057	18.1 (17.5–18.8)^b^
Other^f^	1,936	3.2 (2.9–3.5)	57	2.6 (1.9–3.5)	160	2.8 (2.3–3.3)	1,719	3.3 (3.0–3.6)
**Metropolitan Statistical Area (MSA)**	63,299	100.0 (—)	2,317	100.0 (—)	6,028	100.0 (—)	54,954	100.0 (—)
Urban	25,628	40.4 (39.7–41.1)^a^	572	27.8 (25.5–30.3)	2,388	37.6 (36.1–39.0)^a^	22,668	41.1 (40.3–41.9)^a^
Suburban	24,989	47.1 (46.3–47.8)^a^	796	34.7 (32.3–37.1)	2,345	44.6 (43.1–46.2)^a^	21,848	47.8 (47.0–48.6)^a^
Rural	12,682	12.6 (12.2–12.9)^b^	949	37.5 (35.1–40.0)	1,295	17.8 (16.7–19.0)^b^	10,438	11.1 (10.7–11.5)^b^

Abbreviations: CI, confidence interval; HPV, human papillomavirus; DRA, Delta Regional Authority.

a *P* < .05; value significantly higher than value for similar group in DRA counties; determined by multivariable logistic regression analysis.

b *P* < .05; value significantly lower than value for similar group in DRA counties; determined by χ^2^ test.

c Insurance categories are mutually exclusive.

d Includes Indian Health Service (IHS), Children’s Health Insurance Programs (CHIP), and some private insurers.

e Mixed indicates that a combination of facility types was listed (private, public, hospital, and STD/school/teen clinics) for the adolescent.

f Includes military health care facilities; Special Supplemental Nutrition Program for Women, Infants, and Children (WIC) clinics; and pharmacies.

Unadjusted HPV vaccination initiation coverage estimates among adolescents in the DRA region were significantly lower (54.3%) than in non-Delta states (61.4%) but similar to coverage in Delta areas outside the DRA region (56.2%) (Table 2). These findings persisted after adjusting for sociodemographic and health care–related variables. Despite the difference in coverage among the 3 areas, unadjusted results demonstrated that HPV vaccination initiation coverage followed a similar pattern in each geographic area. In all 3 geographic areas, HPV vaccination initiation coverage was significantly higher among girls, adolescents who were non-Hispanic white, adolescents whose mother had less than a high school education, adolescents whose mother was not currently married, adolescents who had a well-child visit at age 11 or 12, adolescents who had Medicaid (compared with those having private insurance), adolescents who had received a provider recommendation for HPV vaccination, and adolescents who resided in an urban area (Table 2). However, after adjusting for sociodemographic and health care characteristics, the factors that remained significantly associated with HPV vaccination initiation coverage varied by geographic area.

**Table 2:** Unadjusted and Adjusted Logistic Regression Analysis for Vaccination Coverage Estimates (≥1 HPV Dose) Among Adolescents Aged 13–17 Years, by Geographic Area for Selected Sociodemographic Characteristics, National Immunization Survey-Teen, United States, 2015–2017

Characteristic	All Surveyed Adolescents in United States		Mississippi Delta States				Non-Delta States	
			DRA Counties		Delta Areas Outside DRA			
	Unadjusted % (95% CI)^a^	APR, % (95% CI)	Unadjusted % (95% CI)^a^	APR, % (95% CI)	Unadjusted % (95% CI)^a^	APR, % (95% CI)^a^	Unadjusted % (95% CI)^a^	APR, % (95% CI)
**Overall**	60.7 (60.0–61.4)^b^	—	54.3 (51.7–56.8)	Ref	56.2 (54.6–57.8)	1.01 (.95–1.06)	61.4 (60.6–62.2)^b^	1.06 (1.01–1.11)^b^
**Interview year**								
2015	56.1 (54.9–57.4)^b^	0.91 (0.88–0.93)^b^	50.0 (45.8–54.3)^b^	0.96 (0.86–1.08)	51.1 (48.4–53.9)^b^	0.90 (0.84–0.97)^b^	56.9 (55.5–58.3)^b^	0.91 (0.88–0.94)^b^
2016	60.4 (59.2–61.6)^b^	0.95 (0.93–0.98)^b^	55.4 (50.9–59.8)	1.04 (0.93–1.17)	56.5 (53.6–59.2)^b^	0.97 (0.91–1.04)^b^	61.0 (59.7–62.4)^b^	0.95 (0.92–0.98)^b^
2017	65.5 (64.3–66.7)	Ref	57.9 (53.2–62.4)	Ref	61.0 (58.3–63.6)^b^	Ref	66.3 (65.0–67.6)	Ref
**Sex**								
Male	56.1 (55.1–57.1)^b^	0.93 (0.91–0.96)^b^	48.8 (45.3–52.4)^b^	0.91 (0.83–1.00)	51.5 (49.2–53.7)^b^	0.94 (0.88–0.99)^b^	56.9 (55.8–58.0)^b^	0.94 (0.91–0.96)^b^
Female	65.5 (64.5–66.5)	Ref	60.2 (56.5–63.8)	Ref	61.1 (58.9–63.3)	Ref	66.1 (65.0–67.3)	Ref
**Age of adolescent at interview, y**								
13	55.6 (54.0–57.2)^b^	0.86 (0.83–0.89)^b^	51.6 (46.1–57.1)	0.96 (0.83–1.11)	45.6 (42.1–49.1)^b^	0.78 (0.70–0.86)^b^	56.9 (55.1–58.7)^b^	0.86 (0.83–0.90)^b^
14	59.3 (57.7–60.9)^b^	0.89 (0.86–0.93)^b^	53.7 (47.7–59.5)	0.92 (0.79–1.07)	57.0 (53.4–60.6)	0.92 (0.84–1.00)	59.7 (57.9–61.5)^b^	0.89 (0.86–0.93)^b^
15	61.8 (60.2–63.4)^b^	0.94 (0.91–0.98)^b^	57.8 (52.2–63.2)	1.06 (0.93–1.21)	57.4 (53.9–60.8)	0.95 (0.87–1.03)	62.4 (60.6–64.2)^b^	0.94 (0.90–0.97)^b^
16	62.2 (60.7–63.7)^b^	0.95 (0.92–0.98)^b^	55.5 (49.9–61.1)	1.01 (0.88–1.16)	61.5 (58.1–64.8)	1.03 (0.95–1.12)	62.5 (60.8–64.2)^b^	0.94 (0.90–0.97)^b^
17	64.6 (63.0–66.2)	Ref	52.9 (46.9–58.9)	Ref	59.3 (55.6–62.9)	Ref	65.6 (63.9–67.4)	Ref
**Adolescent’s race/ethnicity**								
Non-Hispanic white	55.3 (54.5–56.2)	Ref	46.8 (43.4–50.2)	Ref	50.7 (48.7–52.6)	Ref	56.3 (55.3–57.2)	Ref
Non-Hispanic black	65.3 (63.3–67.2)^b^	1.07 (1.03–1.12)^b^	60.8 (56.2–65.1)^b^	1.03 (0.91–1.15)	65.3 (60.9–69.4)^b^	1.15 (1.05–1.26)^b^	65.7 (63.4–68.0)^b^	1.07 (1.02–1.12)^b^
Hispanic	69.4 (67.5–71.1)^b^	1.15 (1.11–1.20)^b^	69.6 (60.2–77.6)^b^	1.21 (1.01–1.45)	70.4 (66.2–74.3)^b^	1.29 (1.18–1.40)^b^	69.3 (67.4–71.2)^b^	1.14 (1.10–1.19)^b^
Other	62.5 (60.3–64.7)^b^	1.10 (1.06–1.15)^b^	64.5 (54.9–73.0)^b^	1.12 (0.94–1.34)	59.6 (54.1–64.9)^b^	1.13 (1.02–1.25)^b^	62.8 (60.4–65.1)^b^	1.10 (1.05–1.14)^b^
**Mother’s education**								
<High school graduate	71.8 (69.7–73.8)	Ref	65.6 (59.0–71.6)	Ref	64.9 (60.7–68.8)	Ref	72.6 (70.3–74.8)	Ref
High school graduate	61.1 (59.4–62.7)^b^	0.88 (0.84–0.92)^b^	54.2 (48.8–59.5)^b^	0.86 (0.74–1.00)	58.0 (54.4–61.6)^b^	0.97 (0.87–1.07)	61.7 (59.9–63.5)^b^	0.87 (0.83–0.92)^b^
>High school graduate, some college	57.0 (55.5–58.4)^b^	0.82 (0.78–0.86)^b^	54.5 (49.9–59.1)^b^	0.80 (0.69–0.92)^b^	53.9 (50.9–56.9)^b^	0.86 (0.78–0.96)^b^	57.5 (55.8–59.1)^b^	0.82 (0.78–0.87)^b^
College graduate	59.1 (58.0–60.1)^b^	0.87 (0.83–0.92)^b^	49.3 (45.0–53.7)^b^	0.86 (0.74–1.01)	54.3 (51.8–56.7)^b^	0.91 (0.81–1.01)	59.8 (58.7–61.0)^b^	0.87 (0.83–0.92)^b^
**Mother’s marital status**								
Married	58.1 (57.3–59.0)	Ref	46.1 (42.8–49.4)	Ref	52.8 (50.8–54.7)	Ref	59.0 (58.1–60.0)	Ref
Divorced, widowed, or separated	61.8 (60.1–63.4)^b^	1.03 (1.00–1.07)^b^	62.2 (57.0–67.2)^b^	1.23 (1.10–1.38)^b^	58.8 (55.2–62.2)^b^	1.10 (1.02–1.18)^b^	62.1 (60.2–64.0)^b^	1.02 (0.98–1.06)
Never married	70.0 (67.6–72.3)^b^	1.09 (1.04–1.14)^b^	63.4 (56.0–70.3)^b^	1.17 (0.99–1.40)	67.8 (62.3–72.9)^b^	1.11 (0.99–1.24)	70.8 (68.1–73.4)^b^	1.09 (1.03–1.15)^b^
**Mother’s age, y**								
≤34	66.5 (64.1–68.9)	Ref	56.5 (49.3–63.3)	Ref	53.4 (48.2–58.5)	Ref	68.8 (66.1–71.4)	Ref
35–44	60.8 (59.7–61.9)^b^	0.93 (0.89–0.97)^b^	59.3 (55.6–62.8)	1.10 (0.94–1.29)	57.4 (55.0–59.7)	1.05 (0.94–1.17)	61.3 (60.0–62.5)^b^	0.91 (0.86–0.96)^b^
≥45	59.5 (58.5–60.5)^b^	0.93 (0.88–0.97)^b^	46.8 (42.7–50.8)^b^	0.94 (0.78–1.12)	55.6 (53.2–57.9)	1.04 (0.93–1.17)	60.3 (59.2–61.3)^b^	0.91 (0.86–0.96)^b^
**Adolescent had preventive care visit at age 11 or 12**								
Yes	61.6 (60.9–62.3)^b^	1.08 (1.02–1.13)^b^	55.9 (53.1–58.6)^b^	1.12 (0.94–1.34)	57.4 (55.8–59.1)^b^	1.10 (0.97–1.24)	62.3 (61.4–63.1)^b^	1.07 (1.02–1.13)^b^
No	53.8 (51.2–56.3)	Ref	42.3 (34.8–50.2)	Ref	45.6 (40.1–51.2)	Ref	55.2 (52.3–58.0)	Ref
**Income-to-poverty ratio**								
<133%	67.8 (66.5–69.1)^b^	1.01 (0.96–1.06)	63.0 (58.9–66.9)^b^	0.99 (0.84–1.17)	63.4 (60.5–66.1)	1.01 (0.91–1.13)	68.5 (67.0–70.0)^b^	1.01 (0.96–1.06)
133% to <322%	56.2 (54.8–57.5)^b^	0.93 (0.89–0.96)^b^	48.0 (43.1–52.9)	0.84 (0.73–0.98)^b^	50.7 (47.7–53.7)^b^	0.88 (0.81–0.96)^b^	57.2 (55.6–58.7)^b^	0.93 (0.90–0.97)^b^
322% to <503%	55.0 (53.4–56.6)^b^	0.95 (0.92–0.98)^b^	46.3 (40.1–52.5)	0.94 (0.82–1.07)	49.4 (45.9–52.9)^b^	0.89 (0.82–0.97)^b^	55.8 (54.1–57.6)^b^	0.96 (0.92–0.99)^b^
≥503%	60.8 (59.4–62.3)	Ref	49.1 (43.2–55.0)	Ref	59.4 (56.0–62.8)	Ref	61.2 (59.7–62.7)	Ref
**Health insurance coverage^c^**								
Private only	56.9 (56.0–57.8)	Ref	44.1 (40.3–48.0)	Ref	52.8 (50.7–55.0)	Ref	57.7 (56.6–58.7)	Ref
Any Medicaid	67.8 (66.6–69.0)^b^	1.10 (1.06–1.14)^b^	62.9 (59.2–66.4)^b^	1.22 (1.06–1.42)^b^	62.3 (59.7–64.9)^b^	1.06 (0.98–1.16)	68.7 (67.3–70.1)^b^	1.10 (1.05–1.15)^b^
Other^d^	56.8 (54.3–59.3)	1.00 (0.95–1.05)	50.9 (41.2–60.5)	0.93 (0.73–1.19)	55.7 (49.1–62.2)	1.03 (0.90–1.18)	57.1 (54.3–59.8)	1.00 (0.95–1.05)
Uninsured	51.2 (47.4–55.0)^b^	0.94 (0.87–1.02)	44.5 (31.7–58.2)	1.16 (0.85–1.58)	41.9 (33.3–51.1)^b^	0.76 (0.60–0.97)^b^	52.3 (48.1–56.5)^b^	0.95 (0.87–1.03)
**Provider recommended HPV vaccination**								
Yes	71.3 (70.4–72.1)^b^	1.95 (1.86–2.04)^b^	68.0 (64.7–71.1)^b^	2.07 (1.79–2.40)^b^	67.8 (65.9–69.6)^b^	2.02 (1.83–2.22)^b^	71.7 (70.8–72.6)^b^	1.94 (1.84–2.04)^b^
No	36.8 (35.3–38.3)	Ref	32.2 (28.1–36.6)	Ref	33.1 (30.3–36.1)	Ref	37.5 (35.7–39.2)	Ref
**No. of providers**								
1	62.5 (61.5–63.4)	Ref	55.8 (52.3–59.3)	Ref	58.2 (56.1–60.3)	Ref	63.1 (62.1–64.1)	Ref
2 or 3	59.2 (57.8–60.6)^b^	0.93 (0.90–0.96)^b^	52.9 (48.4–57.4)	1.03 (0.93–1.15)	55.0 (52.0–57.9)	0.96 (0.89–1.03)	60.0 (58.4–61.6)^b^	0.92 (0.89–0.95)^b^
≥4	56.7 (54.7–58.6)^b^	0.89 (0.85–0.93)^b^	52.0 (45.3–58.6)	1.00 (0.84–1.18)	51.0 (46.7–55.2)^b^	0.92 (0.83–1.01)	57.4 (55.2–59.6)^b^	0.89 (0.85–0.93)^b^
**No. of physician contacts in the past year**								
None	53.4 (51.3–55.5)	Ref	53.1 (45.4–60.7)	Ref	48.6 (44.0–53.2)	Ref	53.9 (51.6–56.2)	Ref
1	59.2 (57.8–60.6)^b^	1.07 (1.02–1.11)^b^	49.5 (44.3–54.7)	0.86 (0.72–1.02)	54.8 (51.8–57.9)^b^	1.04 (0.94–1.15)	60.0 (58.4–61.5)^b^	1.07 (1.02–1.13)^b^
2 or 3	63.3 (62.2–64.5)^b^	1.12 (1.07–1.17)^b^	54.0 (49.8–58.1)	0.92 (0.79–1.08)	58.2 (55.6–60.7)^b^	1.07 (0.97–1.17)	64.3 (63.0–65.5)^b^	1.13 (1.08–1.18)^b^
≥4	63.6 (62.1–65.0)^b^	1.12 (1.07–1.17)^b^	59.0 (53.9–63.9)	0.97 (0.82–1.14)	59.2 (55.8–62.5)^b^	1.10 (0.99–1.22)	64.3 (62.6–65.9)^b^	1.12 (1.07–1.18)^b^
**Type of facility where vaccinations were obtained**								
All private	60.7 (59.7–61.7)	Ref	56.3 (52.0–60.5)	Ref	57.5 (55.2–59.8)	Ref	61.1 (60.0–62.2)	Ref
All public	60.7 (58.9–62.6)	1.00 (0.96–1.04)	52.7 (47.4–57.9)	0.92 (0.80–1.06)	54.3 (50.4–58.2)	0.97 (0.88–1.06)	62.1 (60.0–64.3)	1.01 (0.96–1.06)
All hospital	64.6 (62.6–66.6)^b^	1.03 (0.99–1.07)	61.9 (52.3–70.6)	1.00 (0.85–1.18)	59.4 (54.2–64.4)	0.97 (0.88–1.08)	65.3 (63.0–67.5)^b^	1.04 (1.00–1.09)
Mixed^e^	61.0 (59.4–62.6)	1.06 (1.02–1.10)^b^	51.3 (46.5–56.1)	0.94 (0.82–1.07)	55.9 (52.5–59.2)	1.02 (0.94–1.10)	62.2 (60.4–64.0)	1.07 (1.03–1.11)^b^
Other^f^	53.6 (49.0–58.1)^b^	0.92 (0.85–1.00)^b^	56.7 (41.1–71.1)	1.15 (0.93–1.44)	44.0 (34.5–53.8)^b^	0.79 (0.64–0.99)^b^	54.4 (49.4–59.4)^b^	0.93 (0.85–1.01)
**Metropolitan Statistical Area (MSA)**								
Urban	66.1 (65.0–67.3)^b^	1.13 (1.10–1.17)^b^	61.7 (56.4–66.7)^b^	1.14 (1.00–1.29)^b^	63.5 (61.0–65.9)^b^	1.17 (1.07–1.28)^b^	66.5 (65.3–67.7)^b^	1.13 (1.09–1.18)^b^
Suburban	58.3 (57.2–59.3)^b^	1.05 (1.02–1.09)^b^	53.6 (49.3–57.8)	1.06 (0.95–1.19)	54.8 (52.3–57.3)^b^	1.11 (1.01–1.21)^b^	58.8 (57.6–59.9)^b^	1.05 (1.00–1.09)^b^
Rural	52.3 (50.8–53.8)	Ref	49.5 (45.4–53.5)	Ref	44.4 (40.9–48.0)	Ref	54.1 (52.3–55.9)	Ref

Abbreviations: APR, adjusted prevalence ratio; CI, confidence interval; DRA, Delta Regional Authority; HPV, human papillomavirus; Ref, reference.

a Percentages are weighted; estimates with 95% CI >20 might not be reliable.

b *P* < .05 by *t* test for comparison with reference group.

c Insurance categories are mutually exclusive.

d Includes Indian Health Service (IHS), Children’s Health Insurance Programs (CHIP), and some private insurers.

e “Mixed” indicates that a combination of facility types was listed (private, public, hospital, and STD/school/teen clinics) for the adolescent and not just one type.

f Includes military health care facilities, Special Supplemental Nutrition Program for Women, Infants, and Children (WIC) clinics, and pharmacies.

In DRA areas, characteristics independently associated with higher rates of HPV vaccination initiation coverage (ie, APR >1 and *P* value <.05) among adolescents were having a mother who was divorced, widowed, or separated compared with a married mother, having Medicaid health insurance compared with having only private insurance, having received a provider recommendation for HPV vaccination compared with no provider recommendation, and residing in urban areas compared with rural areas (Table 2). Lower HPV vaccination initiation coverage (ie, 0 < APR < 1 and *P* value <.05) was found among adolescents whose mothers were high school graduates or had some college compared with mothers having less than a high school education, and those with a household IPR of 133% to less than 322% compared with those having a household IPR of 503% or more.

In the Delta region outside the DRA, factors associated with higher HPV vaccination initiation coverage were identification as non-Hispanic black, Hispanic, or other race/ethnicity compared with adolescents identified as non-Hispanic white; having a mother who was divorced, widowed, or separated, compared with a married mother; receipt of a provider recommendation for HPV vaccination compared with no provider recommendation; and residing in urban or suburban areas compared with rural areas (Table 2). Factors associated with lower HPV vaccination initiation coverage were being male; being aged 13 compared with aged 17; having a mother who was a high school graduate or had some college, compared with a mother having less than a high school education; having a household IPR from 133% to less than 503% compared with those having a household IPR of 503% or more; being uninsured compared with having only private insurance; and having received all vaccinations at an “other” facility compared with an all private facility.

In non-Delta states, factors associated with higher HPV vaccination initiation coverage among adolescents were identification as non-Hispanic black, Hispanic, or other compared with non-Hispanic white; having a mother who had never married compared with a married mother; having received a preventive care visit at age 11 or 12 compared with no visit; having Medicaid health insurance compared with only private insurance; having received a provider recommendation for HPV vaccination compared with no provider recommendation; having had at least 1 physician contact in the past year compared with no physician contact; having vaccinations in a mix of facility types compared with solely in private facilities; and residing in rural areas compared with residing in urban or suburban areas (Table 2).

Outside the Delta, factors associated with lower HPV vaccination initiation coverage were being male compared with female; being aged 13 to 16 compared with 17; having a mother who was a high school graduate compared with a mother with less than a high school education; having a mother aged 35 years or more compared with 34 years or less; having a household IPR of 133% to less than 503% compared with a household IPR of 503% or more; and having 2 or more vaccination providers compared with only 1 (Table 2).

In the DRA, among adolescents without any HPV vaccinations, 49.8% of parents reported a very likely or somewhat likely intent for their adolescent to receive the HPV vaccine in the next 12 months (Table 3). Among parents who did not intend to get their adolescent vaccinated (ie, those who responded not too likely, not likely at all, and not sure or don’t know), the most common reasons for not intending to get the HPV vaccine were that vaccination is not necessary, not having received a recommendation for HPV vaccine from the provider, concerns about vaccine safety or side effects, lack of knowledge about the vaccine, and believing that their adolescents were not sexually active (Table 3). These results were not significantly different from findings in Delta areas outside the DRA or the non-Delta states.

**Table 3:** Survey Responses Among Parents Whose Adolescent Child Had Not Yet Received HPV Vaccination, National Immunization Survey-Teen, United States, 2015–2017^a^

Questions and Responses	All Surveyed Teens in the United States	Mississippi Delta States		Non-Delta States
		DRA Counties	Delta Outside DRA	
**No. of parents who answered question, “How likely is it that [TEEN] will receive HPV shots in the next 12 months?”**	40,929	1,633	4,156	35,140
**Responses**				
Very likely	28.9 (28.1–29.8)	28.2 (25.5–31.1)	28.6 (26.9–30.4)	29.0 (28.1–30.0)
Somewhat likely	24.1 (23.3–24.9)	21.6 (19.2–24.2)	23.6 (22.0–25.3)	24.2 (23.4–25.2)
Not too likely	16.8 (16.1–17.4)	18.2 (16.0–20.7)	17.3 (15.8–18.8)	16.6 (15.9–17.4)
Not likely at all	26.7 (25.9–27.5)	28.5 (25.9–31.3)	26.9 (25.2–28.6)	26.6 (25.7–27.5)
Not sure or don't know	3.5 (3.2–3.9)	3.4 (2.4–4.7)	3.7 (3.0–4.5)	3.5 (3.2–3.9)
**No. of parents who answered open-ended question, “What is the main reason [TEEN] will not receive HPV shots in the next 12 months?”^b^**	19,263	842	2,023	16,398
**Responses**				
Not needed or not necessary	20.6 (19.4–21.9)	20.8 (16.9–25.3)	19.6 (17.1–22.5)	20.8 (19.4–22.2)
Safety concern or side effects	17.3 (16.2–18.3)	15.4 (12.0–19.7)	16.4 (14.1–19.0)	17.4 (16.3–18.6)
Not recommended	13.1 (12.0–14.3)	15.5 (11.9–20.0)	12.7 (10.6–15.2)	13.0 (11.8–14.4)
Lack of knowledge	10.9 (10.0–12.0)	11.9 (8.9–15.6)	12.6 (10.4–15.1)	10.7 (9.6–11.8)
Teen not sexually active	10.3 (9.3–11.4)	9.9 (7.3–13.3)	8.4 (6.8–10.4)	10.6 (9.5–11.8)

Abbreviations: DRA, Delta Regional Authority; HPV, human papillomavirus.

a All values are weighted percentages (95% confidence intervals).

b Responses are from parents and guardians who responded, “not too likely,” “not likely at all,” or “not sure or don’t know” when asked how likely it was that the teen would receive an HPV vaccination in the next year.

## Discussion

The DRA is a subset of the most distressed counties and parishes in 8 states of the southeastern United States. Overall, HPV vaccination coverage inside the DRA region was similar to that in Delta areas outside the DRA but significantly lower than in non-Delta states. Although vaccination coverage was lower in the DRA region, the pattern of coverage was similar to the other 2 geographic areas; however, factors that remained significantly associated with coverage differed after adjusting for demographic characteristics, health insurance, and access to care variables. In the Delta states, factors associated with vaccination coverage included mother’s marital status, mother’s education level, poverty level, any Medicaid health insurance, residence in an urban area, and receiving a provider recommendation for HPV vaccination.

Among adolescents living in the DRA region, HPV vaccination initiation coverage was 19 percentage points higher for those with any Medicaid health insurance, compared with adolescents having private insurance coverage. The higher coverage might likely be because of the availability of vaccines through the VFC (Vaccines for Children) program in the United States (21), which provides vaccines at no cost to eligible children (ie, those without health insurance, who are Medicaid eligible, of American Indian or Alaska Native descent, or whose insurance does not cover the cost of vaccination). Although uninsured children can receive vaccines through the VFC program, vaccination coverage in the DRA region was low (44.5%) but similar to children with private insurance (44.1%). Additional efforts are needed to promote the use of the VFC program among those who are insured. Furthermore, although uninsured children face additional challenges beyond cost to receiving vaccines, understanding challenges to HPV vaccination for privately insured children is also needed.

Although provider recommendation for vaccination was associated with HPV vaccination initiation in all 3 geographic areas, children inside the DRA region were less likely to have received a provider recommendation for HPV vaccination than children outside the DRA region. Previous research has indicated that both the source and manner of recommendation influence parental receptiveness to HPV vaccination (22,23); physicians are a trusted source of vaccination information and could be a crucial influence for increasing HPV vaccination in the DRA region and elsewhere. An announcement that includes a statement that assumes parents are ready to vaccinate results in higher vaccination coverage (22). CDC has developed resources incorporating these communication principles to demonstrate how to give an effective recommendation (24) that might be helpful for clinicians inside the DRA region and elsewhere. Among unvaccinated adolescents, the most common reason their parents did not intend to vaccinate them with the HPV vaccine was the belief that the vaccine was not necessary because their child was unlikely to have initiated sexual activity. This was a prevalent reason across all 3 geographic areas. Recent research has tested and identified effective messages to address these questions and concerns from parents (24). Messages emphasizing cancer prevention were more effective in increasing confidence to vaccinate among parents, whereas messages emphasizing urgency to vaccinate were counterproductive. Effective communication messages to providers are needed to improve their confidence and ability to discuss HPV vaccination with patients. To reach parents and adolescents with limited access to health care providers, engaging partners serving these populations (eg, the Special Supplemental Nutrition Program for Women, Infants, and Children [WIC]) might also be helpful.

Although a higher proportion of adolescents with 2 or 3 providers lived in the DRA region, compared with the other 2 geographic areas, vaccination coverage was not associated with the number of providers among adolescents in the DRA region. Those who have several health care providers might have their medical histories dispersed to multiple providers, and follow-up might be difficult or unlikely. Record scattering, shown to affect vaccination coverage for young children, might also contribute to lower HPV vaccination coverage (25). Encouraging providers to report vaccines they administer to their state immunization information system could help consolidate vaccination records and facilitate timely vaccination decisions during health care encounters.

Thirty-six percent of adolescents in the DRA region received all of their vaccinations at private facilities. Receipt of all vaccinations from private facilities was more common in the other 2 geographic areas. Additional research is needed to determine if this finding results from fewer private facilities operating in the DRA or a reduced likelihood among private facilities in the DRA region to stock and administer HPV vaccines.

This study has several strengths. First, NIS-Teen includes provider-reported vaccination data, which are more reliable than parental recall or vaccination shot cards. Second, multiple years of data were combined to increase sample size and study power to allow detailed analysis of this underserved geographic area. Third, although NIS-Teen was previously limited to households with landline telephones, this data set included cell phone sampling frames as well, which was instrumental in increasing how the data represented the target population.

Our study also had limitations. First, incomplete provider vaccination records and lack of data on community- or county-level factors that might influence HPV vaccination could have limited the scope of this study. Second, after weighting adjustments to mitigate bias from incomplete data in the sample frame and nonresponses, some bias may remain (16). Third, provider recommendation is also subject to recall bias. Finally, some estimates may be unreliable because of the small sample size. Despite these limitations, we believe our findings can raise awareness among providers and policy makers in the DRA region regarding disparities in HPV vaccination coverage, the need for strategies to increase HPV vaccination, and the target populations to consider for enhanced efforts.

Although factors related to HPV vaccine initiation are similar in the 3 areas studied, overall vaccination levels are lower in the Delta (both inside and outside the DRA region). Lower vaccination levels are likely correlated with the unique sociodemographic and health care characteristics of the areas, which are likely also responsible for disparities in HPV vaccination initiation across the 3 geographic areas. In the DRA region, assisting providers in effectively recommending HPV vaccination could be a primary strategy to increase coverage, as recommendations were closely associated with HPV vaccine initiation. Existing resources to help communicate HPV vaccine recommendations might need to be evaluated to ensure cultural appropriateness. To help identify strategies to increase HPV vaccination, additional research is needed to understand the barriers to vaccination in the Delta region, especially differences between uninsured and privately insured adolescents.
